# Circumcision: postoperative complications that required reoperation

**DOI:** 10.1590/S1679-45082018AO4241

**Published:** 2018-07-24

**Authors:** Carolina Talini, Letícia Alves Antunes, Bruna Cecília Neves de Carvalho, Karin Lucilda Schultz, Maria Helena Camargo Peralta Del Valle, Ayrton Alves Aranha, Wilmington Roque Torres Cosenza, Antonio Carlos Moreira Amarante, Antonio Ernesto da Silveira

**Affiliations:** 1 Hospital Pequeno Príncipe, Curitiba, PR, Brazil.

**Keywords:** Phimosis, Child, Circumcision, male/surgery, Circumcision, male/complications, Postoperative complications, Fimose, Criança, Circuncisão masculina/cirurgia, Circuncisão masculina/complicações, Complicações pós-operatórias

## Abstract

**Objective:**

To evaluate post-operative complications of circumcision requiring surgical reintervention.

**Methods:**

Retrospective analysis of medical records of patients submitted to circumcision from May 1^st^, 2015 to May 31^st^, 2016.

**Results:**

A total of 2,441 circumcisions were performed; in that, 1,940 using Plastibell and 501 by the classic technique. Complications requiring surgical reintervention were found in 3.27% of patients. When separated by surgical technique, 3.4% of circumcisions using Plastibell device required reoperation, as compared to 3% of conventional technique (p=0.79). Preputial stenosis was most frequently found in classic circumcision, with statistical significance (p<0.001). Bleeding was more frequent when using Plastibell device, but the difference was not statistically different (p=0.37). Patients’ age was also evaluated to investigate if this variable influenced on the postoperative outcome, but no significant difference was found.

**Conclusion:**

There was no statistically significant difference when comparing complications between the different techniques performed at this hospital. Preputial stenosis was most frequently found in the classic circumcision, while bleeding was more prevalent when using Plastibell device. Patients’ age did not influence in complications.

## RESUMO

**Objetivo:**

Avaliar as complicações pós-operatórias de postectomia que necessitaram de reintervenção cirúrgica.

**Métodos:**

Estudo retrospectivo com análise de prontuários de pacientes submetidos à postectomia entre 1^o^ de maio de 2015 a 31 de Maio de 2016.

**Resultados:**

Foram realizadas 2.441 postectomias no período, sendo 501 utilizando a técnica clássica e 1.940 utilizando o dispositivo Plastibell. Apresentaram complicações que necessitaram reintervenção cirúrgica 3,27% dos pacientes. Quando separados por técnica operatória, 3,4% das postectomias com Plastibell foram reoperadas, comparando com 3% das postectomias convencionais (p=0,79). A estenose de prepúcio foi mais frequentemente encontrada na técnica clássica, com significância estatística (p<0,001). Sangramento foi mais frequente nos casos com uso do Plastibell, porém sem diferença significativa (p=0,37). A idade dos pacientes também foi avaliada para investigar se esta variável influenciou na taxa de complicações pós-operatórias, porém não houve diferença significativa.

**Conclusão:**

Não houve diferença estatisticamente significativa quando comparadas as complicações entre as diferentes técnicas cirúrgicas utilizadas neste serviço. A estenose de prepúcio foi mais frequentemente encontrada nos pacientes operados pela técnica convencional enquanto demonstrou-se tendência a maior sangramento com uso do Plastibell. A idade dos pacientes não influenciou na presença de complicações.

## INTRODUCTION

Circumcision is one of the most ancient surgical procedures, and today is frequently performed by pediatric surgeons. In large American reviews, the circumcision rates in newborns can reach up to 61.1%, and many operations are related to cultural indications, and not only for medical reasons.^1^ A recent Brazilian study evaluated circumcisions performed for medical reasons during a 27-year period, in the public healthcare system, and demonstrated that 2.1% of all boys aged 1 to 14 years were circumcised. When age was limited to 1 to 4 years, it was noted that 1.1% of these children were circumcised within this period.^2^

The benefits of surgery include prevention of urinary infection and pyelonephritis, decrease in penile cancer rates, as well as a reduction in sexually transmitted diseases. Nevertheless, like other procedures, it is not exempt from complications.

The complication rates depend on multiple factors, including anatomical anomalies, clinical comorbidities, surgical technique used, and age of patients.^1^ Bleeding is the most common complication of circumcision, with an incidence of up to 1% in recent retrospective studies. Meticulous attention to hemostasis during the procedure, and adequate compression on the skin suture can prevent this complication in most cases. Even so, there may be dislodgment of clots.^1^ In general, late complications are associated with remaining skin inclusions and the incomplete removal of the prepuce, which can result in wound contraction and scarring of the distal portion of the prepuce, leading to its stenosis. The fibrotic ring may, then, result in true phimosis, requiring reoperation in about 2% of cases.^1^Severe complications are rare.^3^ Some pediatric urologists consider that adolescent patients have a greater risk of postoperative bleeding, but this association has not been duly confirmed by literature.^4^

## OBJECTIVE

To evaluate circumcision complications and compare them with the different surgical techniques used, and the influence of patient age in the postoperative complications.

## METHODS

This was a retrospective study, by means of the analysis of medical records of 2,441 patients submitted to circumcision between May 1^st^, 2015, and May 31^st^, 2016, at the *Hospital Pequeno Príncipe*, in Curitiba, State of Paraná, Brazil. The present study was approved by the Ethics Committee of the organization, official opinion number 1.602.963, CAAE: 56993816.0.0000.0097.

Two different surgical techniques were used, and patients were divided into two groups. The choice was based on the personal opinion and experience of each surgeon. The sample included patients operated on by 19 different surgeons, 6 of whom were residents in pediatric surgery.

All patients were operated on based on medical indication, and no newborn underwent surgery. Some similarities were noted among the two groups: blockage of the dorsal penile nerve was performed in all patients under general anesthesia, and no prophylactic antibiotic was used.

The first technique, performed on 501 patients, consisted of classic circumcision. The redundant prepuce was retracted, the adhesions to the glans were released, and after skin excision, hemostasis was performed with an electrocautery. Approximation of the skin and mucosal borders was done with absorbable suture (Catgut 5-0).

The second technique consisted of the use of Plastibell. The redundant prepuce was pulled upwards, and the device was positioned between the prepuce and the glans. The size of the ring was chosen according to the size of the glans. A non-absorbable suture was tied around the device, and the distal prepuce was excised. No bandaging was used, and in the immediate postoperative phase, topical neomycin was used. This group consisted of 1,940 patients.

All cases were analyzed, and the sample consisted of 80 patients who presented with complications requiring surgical reintervention. The data collected was name, age on the day of surgery, surgical technique used, complications, and surgical treatment given after complication.

The data were tabulated using Microsoft Office Excel^®^ and analyzed using Fisher’s exact test. A p value <0.05 was considered significant.

## RESULTS

**Figure 1:**
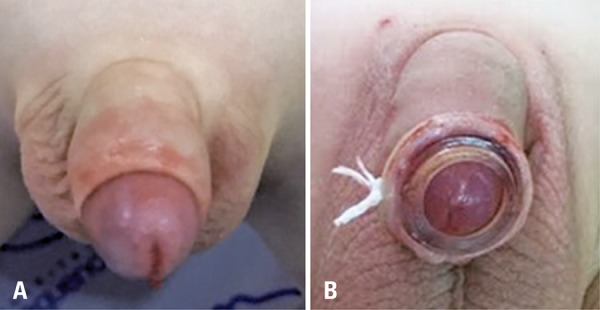
Techniques for circumcision

Of the total 2,441 circumcisions performed at the service during the period evaluated (Figure 1), 80 (3.27%) of them presented with complications, requiring surgical reintervention.

(A) Conventional circumcision. (B) Circumcision with plastic ring.

Among all patients with complications, 18.8% were submitted to classic circumcision and 81.2% were operated on using the plastic ring. The complication rate was 3% in the conventional circumcisions and 3.4% in the circumcisions done with Plastibell. The value was 0.79 according to the test of proportions, demonstrating there was no significant difference between the two proportions.

**Figure 2:**
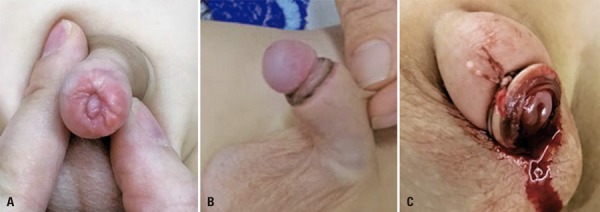
Postoperative complications

We found penile stenosis in 22.8% of cases, bleeding in 32.9%, paraphimosis caused by dislodgement of the plastic ring in in 41.8%, hematoma at the site of anesthetic puncture in 1.2%, and operative wound infection associated with retention of the Plastibell by the prepuce in 1.2% (Figure 2).

(A) Preputial stenosis after classic circumcision. (B) Paraphimosis due to dislodgment of the plastic ring. (C) Hematoma after circumcision with plastic ring.

Two patients presented with complications more than once. The first was submitted to classic circumcision and evolved with local bleeding, needing to be reoperated by conventional technique, and progressing with stenosis of the foreskin, which was corrected with a new classic circumcision. The second patient was initially submitted to circumcision with Plastibell, and evolved with local bleeding and a reoperation by the classic technique; during evolution, the patient presented with preputial stenosis, which was corrected by a new circumcision with the classic technique. Four patients who presented with postoperative preputial stenosis, also presented with balanoposthitis, requiring treatment with antibiotics and reoperation.

When the complications were evaluated separately, evolution with preputial stenosis was noted more frequently in patients submitted to the classic circumcision, with a statistically significant difference when compared to the use of the plastic ring (p<0.001). The presence of bleeding was most prevalent in cases in which the ring was used (23 cases *versus* 3 cases). Considering that no significant difference was demonstrated (p=0.37), we can only affirm that there was a greater tendency towards bleeding with the use of Plastibell (Table 1).

**Table 1:** Complications of circumcision according to surgical technique

Complication	Conventional n (%)	Plastibell n (%)	p value
	Plastibell	6 (0.3)	<0.001
	Valor de p	23 (1.2)	0.37
Preputial stenosis	12 (2.4)	6 (0.3)	<0.001
Bleeding	3 (0.6)	23 (1.2)	0.37
Paraphimosis (dislogdement of the ring)	0	33 (1.7)	
Others	0	2 (0.1)	

Proportion test. p value <0.05 indicates significant difference.

The age of patients varied between 10 months and 13 years, with a mean of 5.27 years and a median of 4 years. Complications were also analyzed according to age range, but there was no significant difference among the groups of complications as to mean age.

Regarding the surgical treatment chosen after complication, we found 30% of ring removal, 33.7% of ring removal followed by hemostasis and suture, 17.5% of reoperation by conventional technique, 6.2% of patients resolved merely with hemostasis by cautery, 3.8% needed hemostasis and re-suture, and 1.2% had the ring removed and still required hemostasis with the cautery. The patient who presented with hematoma at the anesthetic puncture site was submitted to local exploration and hemostasis, while the patient with wound infection and ring retention was submitted to removal of the device and tissue debridement.

## DISCUSSION

Approximately 60% of male newborns in the United States are circumcised at birth,^5^ and it is estimated that out of the remaining 40%, 4% will be circumcised before reaching 15 years of age.^6^ Recent studies also considered that there are so many benefits of circumcision, that in newborns it should be a part of public health policies.^7^ There are few data evaluating the rates of complications and the need for reoperation after elective procedures. Early complications include bleeding, hematoma, infection, inflammation, and fever. Some complications can be related to anesthesia. The most traumatic complications for parents and patients are hemorrhages that require emergency surgical exploration to control bleeding and remove hematoma.^4^Recent studies have demonstrated reoperation rates as low as 0.1%,^3^ but other investigations showed similar data to those found in this study – about 3 to 4.5%.^(8,9^

This study demonstrated there is no difference in the reoperation rates, regardless of age of patients. Children aged 12-18 years are 0.77-fold less likely to be readmitted at the emergency department within 7 days after the operation, although they are 1.91-fold more likely to require a second surgical procedure.^4^A recent metanalysis also showed greater complications in the groups of older patients.^8^

Plastibell was initially used to perform circumcisions in 1956. Since then, its use has been increasingly more widespread and frequent with satisfactory success rates, although there are reports of complications associated with its use.^10^ One large study published in 2013 described 119 cases of circumcision utilizing the plastic ring, and demonstrated immediate complications in 7 (5.9%) patients. Three of them presented with hemorrhage and need for immediate surgical reintervention (conversion to conventional surgery). When long-term complications were evaluated, we noted 32 cases, the majority of little clinical significance (adherences between the glans and prepuce, fibrotic scar, preputial edema, and one case of urethral meatus stenosis). Complications directly related to the use of the ring occurred in 6 (5.4%) patients – five of them with significant postoperative pain, and one who presented Plastibell retention by the foreskin, requiring manual removal. The complication rates did not show correlation with the size of the ring used, and were similar in the different age ranges.^10^ The incidence of early complications with the use of the plastic device reported by Bastos Netto et al., was similar to that of the conventional technique, and corroborates data of our study.^10^

Several studies described bleeding^10,11^ as the most common early complication with the use of Plastibell, and the rates varied from 2.5 to 4%. In general, bleeding results from the inappropriate ligature of the cord that holds the plastic ring, predisposing foreskin retraction and dislodgement of the ring.^10^ In this study, bleeding was the second most frequent complication of Plastibell use, following paraphimosis caused by dislodgement of the plastic ring – a complication that has not been previously analyzed in literature, but which, in this study, was responsible for the highest rate of reoperation among all patients.

Four patients from this study presented with postoperative balanoposthitis associated with preputial stenosis, the latter considered a late complication associated primarily with the greater quantity of foreskin that is maintained, and with the process of local scarring. Previous literature demonstrated the incidence of this infectious complication can be as high as 15%^9^ of cases. Some authors^9,10^ recommended surgical revision as soon as possible in patients with preputial stenosis or redundant prepuce, in order to avoid this complication.

Some severe complications with potential anatomical, functional, and psychological sequelae have already been reported, such as amputation of the glans, lesion of the corpus cavernosum, ablation of the penis, iatrogenic micropenis, and penile amputation.^12^ Assessing the complications using the Clavien-Dindo classification^13^ for surgical complications, the classes IIIa and IIIb (complications with need for surgical reintervention) were emphasized in this study. No cases of class IV (life-threatening complications) were found. Another recently published study proposed a classification of circumcision complications by separating them into five different grades: I for skin problems; II, isolated urethral lesion; III, amputation of the glans; IV, lesion of the corpus cavernosum; and V, total loss of the phallus.^14^ According to this recent classification, this article did not present with complication among those considered most severe.

As to the limitations of the study, we highlight the fact of being conducted at single center, where different surgeons were involved in the procedures, including residents under training, which could have influenced the clinical progress in some patients.

## CONCLUSION

There was no statistically significant difference when comparing complications among the different surgical techniques used at this service. Preputial stenosis was more often observed in patients operated on by the conventional technique, whereas bleeding tended to be more frequent with the use of Plastibell. Paraphimosis due to dislodgement of the plastic ring was a frequent complication that required surgical reintervention. The age of patients did not influence in presence of complications.
